# Comparison of Clinical Characteristics and Outcomes of COVID-19 Between Young and Older Patients: A Multicenter, Retrospective Cohort Study

**DOI:** 10.7759/cureus.21785

**Published:** 2022-01-31

**Authors:** Chukwuemeka Umeh, Kimberly Watanabe, Laura Tuscher, Sobiga Ranchithan, Rahul Gupta

**Affiliations:** 1 Internal Medicine, Hemet Global Medical Center, Hemet, USA; 2 Medicine and Surgery, American University of Antigua, St. John’s, ATG

**Keywords:** icu, mechanical ventilation, mortality, young patients, elderly, covid-19

## Abstract

Background

Outcomes of coronavirus disease 2019 (COVID-19) have been reported to be different in the young and elderly populations. However, previous studies examining these characteristics and differences in outcomes between the two groups had a small sample size. Therefore, in this study, we evaluate the differences between young and elderly patients using a large multicenter dataset.

Methodology

We conducted a retrospective study of 1,116 consecutive COVID-19 patients admitted to two hospitals in southern California in the United States between March 2020 and March 2021. In this study, we categorized patients into two age groups: less than 65 years and 65 years and above. Finally, Kaplan-Meier and backward selection Cox multivariate regression analyses were done using mortality as the dependent variable.

Results

Our analysis showed increased survival in patients aged less than 65 years compared to those aged 65 years or above (p < 0.001). Furthermore, in patients aged 65 years and above, age (hazard ratio (HR) = 1.05; p < 0.001), C-reactive protein (CRP) (HR = 1.05; p < 0.001), and bradycardia (HR = 2.1; p < 0.001) were significantly associated with mortality. Similarly, CRP (HR 1.05; p = 0.02) was significantly associated with mortality in patients aged less than 65 years. However, contrary to many studies, being male (HR = 0.46; p = 0.002) was protective against mortality in patients aged less than 65 years.

Conclusions

Our study showed that the predictors of mortality in COVID-19 patients differed by age group. While age, CRP, and bradycardia were associated with mortality in those aged less than 65 years, only CRP was associated with mortality in those aged 65 years and above.

## Introduction

The severe acute respiratory syndrome coronavirus 2 (SARS-CoV-2), commonly known as the coronavirus disease 2019 (COVID-19), has infected over 260 million people and taken the lives of over five million individuals worldwide as of early December 2021 [1]. Since its discovery in December 2019, concerns over the COVID-19 global health transmission have led to the need to diagnose and control the highly infectious disease quickly. Epidemiological and clinical data from infected individuals have been used to develop both diagnostic and therapeutic procedures. However, the clinical characteristics of the COVID-19 virus range in severity from asymptomatic cases to those causing death. During the study period, there were several common symptoms that individuals infected with COVID-19 frequently experience, such as cough, fever, myalgia, shortness of breath, or difficulty breathing. Additionally, many people experience clinical symptoms, such as weakness, loss of taste or smell, fatigue, nausea, vomiting, and diarrhea [2]. These symptoms typically appear between two and fourteen days after an individual is exposed to the virus [3]. The wide range in symptoms of COVID-19 and the fluctuation in symptom timeline have shown how different it may present from one individual to the next.

With the wide range of clinical characteristics and outcomes within infected individuals, there have been differences between young and elderly patients. Among individuals infected with COVID-19, those with chronic illnesses, such as diabetes, cardiovascular, pulmonary, and kidney disease, have been more severely affected [4]. Other vulnerable groups of individuals include chronic smokers and immunocompromised patients, including individuals on chronic immunosuppressants with a weakened immune system [4]. These comorbidities are disproportionally seen more in elderly patients than in younger and healthier patients. Moreover, the mortality of elderly patients, their susceptibility to severe illness, and admission to the intensive care unit (ICU) are higher compared to younger patients [5]. However, several studies examining the characteristics and differences in outcomes between these age groups had a small sample size. Therefore, in this study, we aimed to evaluate the differences between young and elderly patients using a large multicenter dataset.

## Materials and methods

We conducted a retrospective study of 1,116 consecutive COVID-19 patients admitted to two hospitals in southern California in the United States between March 2020 and March 2021. The study included all patients with a COVID-19 infection confirmed through a positive polymerase chain reaction nasopharyngeal swab. We extracted relevant de-identified patient data from electronic medical records, including age, sex, race, marital status, comorbidities, such as hypertension and heart failure, laboratory results on admission, admission date, discharge date, medications administered in the inpatient setting, and disposition at discharge. In addition, we categorized patients into the following two age groups: less than 65 years and 65 years and above. Furthermore, we defined bradycardia as a sustained heart rate of <60 beats per minute on two separate occasions, a minimum of four hours apart during the hospitalization. Our primary outcome was in-hospital mortality by age group and factors that affect mortality. The secondary outcomes included length of hospital stay, admission to the ICU, and the need for invasive mechanical ventilation by age group.

Univariate analysis of study variables was done using means and percentages. We compared the characteristics of the different age groups using the chi-square test for categorical variables and t-test for continuous variables, with a p-value of 0.05 considered significant. We used the Kaplan-Meier test to compare survival between young and elderly patients. Finally, we performed a backward selection Cox multivariate regression analysis using mortality as a dependent variable. Initially, we included biologically plausible or statistically significant variables in the bivariate analysis, such as patients’ age, sex, race, body mass index (BMI), comorbidities, C-reactive protein (CRP), as independent variables in the multivariate model. The effect was expressed in terms of hazard ratio (HR) with a 95% confidence interval (CI). Statistical analysis was done using SPSS version 27 (IBM Corp., Armonk, NY, USA). The study was approved by the WIRB-Copernicus Group institutional review board (approval number: 13410516).

## Results

Characteristics of the different age groups

A total of 1,116 patients were admitted with COVID-19 during the study period. Of these, 470 (42%) were aged less than 65 years and 646 (58%) were 65 years and older. The mean age of those aged less than 65 was 49 years, while it was 78 years for those aged 65 years and above. The BMI was significantly higher in the younger age group than the elderly group (p < 0.001). Similarly, CRP (p = 0.024), D-dimer (p < 0.001), and troponin (p = 0.001) were significantly higher in the elderly group than the younger age group (Table 1).

**Table 1:** The characteristics of patients in the two age groups. BMI: body mass index; CRP: C-reactive protein; LDH: lactate dehydrogenase; HR: heart rate

Variable	All patients (n = 1,116 )	Age <65 years (n = 470)	Age ≥65 years (n = 646)	P-value
Age, years (mean (range))	65.52 (19–101)	49.07 (19–64)	77.5 (65–101)	<0.001
BMI, kg/m^2^ (mean (range))	30.78 (14.72–83.13)	33.91 (17.26–83.13)	28.51 (14.72–66.07)	<0.001
CRP	9.02 (0.04–31.22)	8.46 (0.04–31.02)	9.45 (0.05–31.22)	0.024
LDH	405.71 (87–10,543)	387.68 (101–3,279)	419.41 (87–10,543)	0.361
D-dimer	1,330.13 (135–5,250)	1,089.29 (150–5,250)	1,504.41 (135–5,250)	<0.001
Ferritin	800.14 (5.1–51,813)	741.21 (5.1–40,280)	842.81 (15.1–51,813)	0.617
Troponin	0.21 (0.04–18.42)	0.10 (0.04–2.43)	0.28 (0.04–18.42)	0.001
Gender				
Female	541 (48.5%)	241 (51.3%)	300 (46.4%)	0.110
Male	575 (51.5%)	229 (48.7%)	346 (53.6%)	
Race				
White	911 (81.6%%)	379 (80.6%)	532 (82.4%)	0.757
Black	80 (7.2%)	35 (7.4%)	45 (7.0%)	
Others	125 (11.2%)	56 (11.9%)	69 (10.7%)	
Bradycardia (HR <60)	376 (33.7%)	147 (31.3%)	229 (35.4%)	0.145
Severe bradycardia (HR <50)	77 (6.9%)	29 (6.2%)	48 (7.4%)	0.412
Coronary artery disease	206 (18.5%)	51 (10.9%)	155 (24.0%)	<0.001
Chronic obstructive pulmonary disease	157 (14.1%)	39 (8.3%)	118 (18.3%)	<0.001
Heart failure	194 (17.4%)	57 (12.1%)	137 (21.2%)	<0.001
Chronic kidney disease	227 (20.3%)	73 (15.5%)	154 (23.8%)	0.001
Acute kidney injury	294 (26.3%)	94 (20.0%)	200 (31.0%)	<0.001
Hypertension	674 (60.4%)	216 (46.0%)	458 (70.9%)	<0.001
Diabetes mellitus	499 (44.7%)	201 (42.8%)	298 (46.1%)	0.264

There was no difference by sex (p = 0.11) and race (p = 0.76) between the younger and elderly age groups. However, coronary artery disease (p < 0.001), chronic obstructive pulmonary disease (p < 0.001), heart failure (p < 0.001), chronic kidney disease (p = 0.001), acute kidney injury (p < 0.001), and hypertension (p < 0.001) were significantly higher in the elderly than the younger age groups (Table 1).

Primary and secondary outcomes of interest

**Figure 1:**
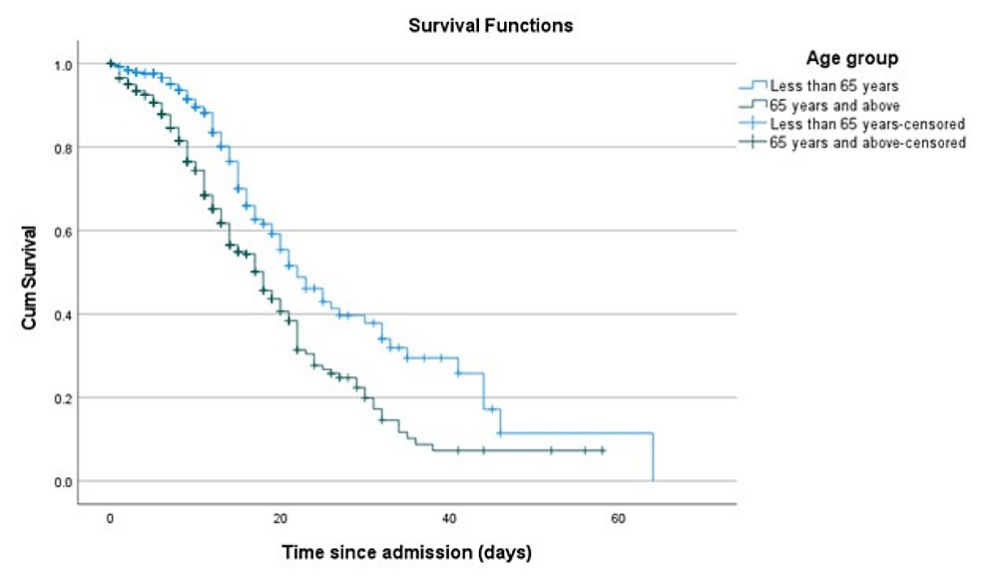
The Kaplan-Meier analysis showing mortality in the two age groups.

The elderly patients were significantly more likely to die than the younger patients (p < 0.001) (Figure 1, Table 2). While 18% of younger patients died, 33% of the elderly died. Additionally, the Kaplan-Meier analysis showed increased survival in patients aged less than 65 years compared to those aged 65 years or above (p < 0.001) (Figure 1). However, there was no difference in ICU admission (p = 0.86), use of invasive mechanical ventilation (p = 0.73), and length of hospital stay (p = 0.91) for the younger and elderly patients (Table 2).

**Table 2:** The primary and secondary outcomes of interest. ICU: intensive care unit

Variable	All patients (n = 1,116 )	Age <65 years (n = 470)	Age ≥ 65 years (n = 646)	P-value
Primary outcome				
In-hospital mortality	295 (26.4%)	84 (17.9%)	211 (32.7%)	<0.001
Secondary outcomes				
ICU admission	238 (21.3%)	99 (21.1%)	139 (21.5%)	0.855
Use of invasive mechanical ventilation	207 (18.5%)	85 (18.1%)	122 (18.9%)	0.734
Length of stay, days (mean (range))	9.03 (0–64)	9.06 (0–64)	9.01 (0–58)	0.911

Predictors of mortality in the different age groups

According to the Cox multivariate regression analysis for mortality in patients aged less than 65 years, being male (HR = 0.46; p = 0.002) and ICU admission (HR = 0.16; p ≤ 0.001) were protective against mortality. Conversely, CRP (HR = 1.05; p = 0.02) was significantly associated with mortality. BMI was also associated with mortality (HR = 1.02; p = 0.066); however, this was not statistically significant (p = 0.05) (Table 3).

**Table 3:** Cox multivariate regression analysis of predictors of mortality for age less than 65 years. SE: standard error; df: degree of freedom; Sig.: significance; HR: hazard ratio; CI: confidence interval; ICU: intensive care unit; BMI: body mass index; CRP: C-reactive protein

	B	SE	Wald	df	Sig.	HR	95.0% CI for HR	
							Lower	Upper
Gender (male)	-0.784	0.250	9.834	1	0.002	0.457	0.280	0.745
ICU admission	-1.807	0.367	24.293	1	0.000	0.164	0.080	0.337
BMI	0.022	0.012	3.374	1	0.066	1.022	0.999	1.046
CRP	0.044	0.019	5.335	1	0.021	1.045	1.007	1.084

According to the Cox multivariate regression analysis for mortality in patients aged 65 years or older, ICU admission (HR = 0.34, p ≤ 0.001) was protective against mortality. Conversely, age (HR = 1.05; p < 0.001), CRP (HR = 1.05; p < 0.001), and bradycardia (HR = 2.1; p < 0.001) were significantly associated with mortality (Table 4).

**Table 4:** Cox multivariate regression analysis of predictors of mortality for age 65 years or greater. SE: standard error; df: degree of freedom; Sig.: significance; HR: hazard ratio; CI: confidence interval; ICU: intensive care unit; CRP: C-reactive protein

	B	SE	Wald	df	Sig.	HR	95.0% CI for HR	
							Lower	Upper
Age	0.044	0.010	17.652	1	0.000	1.045	1.024	1.066
ICU admission	-1.084	0.181	35.871	1	0.000	0.338	0.237	0.482
CRP	0.045	0.013	12.436	1	0.000	1.046	1.020	1.072
Bradycardia	0.761	0.165	21.221	1	0.000	2.139	1.548	2.957

## Discussion

We studied the differences in COVID-19 outcomes between young and elderly patients. Our primary outcome was in-hospital mortality and secondary outcomes were hospital length of stay, ICU admission, and invasive mechanical ventilator use. Similar to previous studies, our study showed that older patients were more likely to die from COVID-19 than younger patients [4,5]. However, the predictors of mortality were different in the two age groups. While increasing age, high CRP, and bradycardia were associated with mortality in the elderly, elevated CRP was associated with mortality in the younger age group. CRP is an inflammatory marker primarily synthesized in liver hepatocytes and traditionally utilized as a marker of infection, inflammation, and cardiovascular events. CRP was predictive of mortality in both the young and elderly in our study [6]. High CRP levels have been noted in COVID-19 patients and have been associated with severe COVID-19, the need for ICU-level care [7], and in-hospital mortality [4,8,9]. However, our study found no differences in hospital length of stay, ICU admission, and ventilator use between the two age groups.

Unlike in the younger population, increasing age was predictive of in-hospital mortality in the elderly. COVID-19 patients aged 65 years or older have markedly higher COVID-19 in-hospital mortality rates than younger patients [10]. However, this difference in COVID-19 mortality between the elderly and young is less prominent in low- and middle-income countries than in high-income countries [11]. A possible explanation for the increase in mortality with age is that comorbidities such as hypertension, coronary artery disease, and diabetes increase with age. Additionally, these comorbidities have been associated with increased COVID-19 mortality [10]. Furthermore, the elderly are more vulnerable to infections due to impaired innate and adaptive immune responses called immunosenescence. This increases the likelihood of a dysfunctional immune response in COVID-19, leading to a cytokine storm and increased mortality [12-15]. Moreover, the elderly have an aging immunity characterized by chronic subclinical systemic inflammation without overt disease called inflammaging. With the concurrent inflammation from COVID-19, inflammaging has been suggested to contribute to increased mortality in the elderly [12-15]. Finally, elderly patients are more likely to be frail than younger patients, and frailty has been associated with increased mortality in COVID-19 patients [16].

Further, unlike in the young, bradycardia was predictive of mortality in the elderly. In previous studies, bradycardia has been associated with increased mortality; however, its mechanism remains unclear [17-19]. One proposed mechanism is that the cytokine storm produced by severe SARS-CoV-2 infection directly affects the sinoatrial node leading to bradycardia [20]. Previous studies have also reported that bradycardia in COVID-19 patients is not associated with hypoxia, myocardial ischemia, or medications that induce bradycardia [17,18]. Our study did not find any difference in the incidence of bradycardia in young and elderly patients (p = 0.15). Thus, the reason why bradycardia was only predictive of in-hospital mortality in the elderly in our study remains unclear.

Although increased BMI has also been reported as a predictor of mortality in previous studies, it only predicted mortality in younger patients in our study. However, this was not statistically significant (p = 0.05). Earlier studies have reported that obesity increases the risk of death from COVID-19, especially in the younger population [21,22]. Individuals with obesity are at a higher risk of contracting COVID-19, being hospitalized from COVID-19, ICU admission, and death from COVID-19 [23]. The mechanisms responsible for greater COVID-19 severity and mortality in individuals with obesity remain unclear. However, several possible mechanisms have been suggested as contributory to increased severity in obese patients. These include metabolic dysfunction, innate and adaptive immune response impairment, and adipose inflammation [23]. Consequently, COVID-19 vaccines can potentially be less effective in obese patients due to a weakened immune response [23].

Surprisingly, being male was protective against mortality in patients aged less than 65 years in our study. However, contrary to our findings, many studies have reported that the risk of dying from COVID-19 is higher for men than for women in almost all age groups [24,25]. It is believed that more robust innate immune responses and more risky health behaviors place men at a greater risk of death from COVID-19 [26,27]. However, the reason for increased mortality in young females in our study is unclear. One possibility might be the differences in our community’s socioeconomic status, race, and health behaviors of young females and males.

Our study has some limitations. First, this is a retrospective observational study, and there may have been unadjusted confounders affecting the study outcomes. Second, the findings of our study are only generalizable to hospitalized COVID-19 patients. Third, we did not have information on some patients’ baseline status that may affect mortality and prognosis, such as frailty status, malnutrition, and dementia. Fourth, we did not have information on the patients’ baseline heart rates prior to admission with COVID-19 and cannot differentiate bradycardia caused by COVID-19 from baseline bradycardia prior to COVID-19. Finally, post-discharge follow-up mortality data were not available, and survival data were limited to patients’ stay in the hospital.

## Conclusions

Our study showed that the predictors of mortality in COVID-19 patients differed by age group. While age, CRP, and bradycardia were associated with in-hospital mortality in those aged less than 65 years, only CRP was associated with in-hospital mortality in those aged 65 years and above. In addition, COVID-19 in-hospital mortality was higher in the elderly than in younger patients. Our study also found no difference in hospital length of stay, ICU admission, and invasive mechanical ventilator use between the two age groups.
